# Emergence of human-like H3N2 influenza viruses in pet dogs in Guangxi, China

**DOI:** 10.1186/s12985-015-0243-2

**Published:** 2015-02-03

**Authors:** Ying Chen, Yan-Ning Mo, Hua-Bo Zhou, Zu-Zhang Wei, Guo-Jun Wang, Qing-Xiong Yu, Xiong Xiao, Wen-Juan Yang, Wei-Jian Huang

**Affiliations:** College of Animal Science and Technology, Guangxi University, No.100 Daxue Road, Nanning, 530004 People’s Republic of China; Huabo Pet Medical Center, Yufeng District, No.342 Liushi Road, Liuzhou, 545005 People’s Republic of China; Department of Microbiology, Mount Sinai School of Medicine, New York, NY 10029 USA

**Keywords:** Pet dogs, H3N2 subtype, Human-like influenza viruses

## Abstract

**Background:**

After the 1968 H3N2 pandemic emerged in humans, H3N2 influenza viruses continuously circulated and evolved in nature. An H3N2 variant was circulating in humans in the 1990s and subsequently introduced into the pig population in the 2000s. This virus gradually became the main subtype of swine influenza virus worldwide. However, there were no reports of infections in dogs with this virus.

**Findings:**

In 2013, 35 nasal swabs from pet dogs were positive for Influenza A virus by RT-PCR. Two viruses were isolated and genetically characterized. In the phylogenetic trees of all gene segments, two H3N2 canine isolates clustered with Moscow/10/99 and most H3N2 swine influenza viruses. These results indicated that two H3N2 CIVs possessed high homology with human/swine influenza viruses, which at the same time exhibited some amino acid substitutions in NA, polymerase basic protein 1 (PB1), and nucleoprotein (NP), which probably were related to the interspecies transmission.

**Conclusions:**

These two viruses share the highest homology with swine H3N2, Moscow/99-like viruses, which indicated that these viruses might originate from swine viruses.

Influenza A viruses circulate worldwide and are endemic in multiple species from birds to mammals [1]. Historically, dogs have been infected with different subtypes of influenza viruses. After the interspecies transmission of influenza H3N8 viruses from horses to dogs was reported in 2005, avian-origin H5N1, H3N2, H5N2 and H9N2, pdm09 H1N1 human, and novel H3N1 influenza viruses with pdm/09 internal genes were also isolated from dogs [2-8]. Recently, a serological survey proved that dogs could be infected with human influenza viruses, and different subtypes of influenza viruses even coexist in dogs [9-11]. Infection of dogs with human H3N2 viruses has been reported [12-14], however, there was a lack of virological evidence about seasonal human H3N2 influenza viruses in dogs. Here, we present the results of genetic and phylogenetic characterization of H3N2 canine influenza viruses (CIVs) isolated in 2013 in Guangxi, China. Genetic analysis demonstrated that human-like H3N2 swine influenza viruses appeared in pet dogs. It provides further evidence that dogs can be regarded as intermediate hosts and can play an important role in influenza ecology.

During April to November 2013, 261 nasal swabs and 315 blood samples were collected from different pet hospitals, including Nanning, Yulin, Liuzhou, Hechi, Baise, Qinzhou, Wuzhou, Pinxiang in Guangxi Zhuang Autonomous Region (Guangxi) of China. Some of these pet dogs showed similar clinical signs of coughing, sneezing, nasal discharge, and fever (>39.5°C), and others had no respiratory syndrome (Table 1). The nasal swab samples were first subjected to M gene specific RT-PCR, as described by Fouchier *et al.* [15]. The positive samples were treated by 200 U/ml penicillin, 200 mg/ml streptomycin and 100 μg/ml gentamicin and centrifuged at 2000 rpm for 5 min and filtered through a 0.45-μm pore size filter (Millipore, Bedford, MA) for isolation of viruses in MDCK cells. Although 35 of 261 nasal swab samples were positive for influenza viruses according to RT-PCR, the isolation of CIVs was difficult, and depended on the adaptation of the viruses to MDCK cells. After six passages in cell culture, two CIVs were isolated and identified as H3N2 subtype whose hemagglutinin (HA) titer against avian erythrocytes reached 1:128 and 1:256, respectively. They were named as A/canine/Guangxi/L1/2013(Ca/GX/L1/2013) and A/canine/Guangxi/L2/2013 (Ca/GX/L2/2013), respectively.

**Table 1:** **Description of two H3N2 canine influenza viruses from Guangxi in 2013**

**Species**	**Dog residence**	**Date**	**Sex**	**Age**	**Clinical signs**	**Isolation** ^**a**^
English sheepdog	Liuzhou	5/3/2013	Female	2 months	gastroenteritis, diarrhea, Canine Parvovirus Virus (CPV) positive	A/canine/Guangxi/L1/2013(H3N2)
Samoyed	Liuzhou	5/10/2013	Male	14 months	Cough, sneeze, nasal discharge, low appetite, depression; body temperature:39.5°C	A/canine/Guangxi/L2/2013(H3N2)

^a^The sequence for two strains were shown in GenBank with the accession numbers KJ013179-KJ013194.

For characterizing the gene segments of the isolated viruses, total virus RNA was extracted using RNAiso Plus (TAKARA, Dalian, China). Reverse transcription was carried out under standard conditions with Uni12 primer (5’-AGCAAAAGCAGG-3’). Eight pairs of primers [16] were used to amplify gene segments for sequencing. PCR products were purified with E.Z.N.A. Gel Extraction Kit (USA) and sequenced by the method of Sanger. The nucleotide sequences showed the highest degree of similarity with the HA sequences of the swine H3N2 subtype (99.9%) and with the NA sequences of the swine H1N2 subtype (99.7%), respectively (Table 2). Therefore, the two CIVs were determined to belong to the H3N2 subtype. The entire genome of the two isolates was deposited in GenBank under accession numbers KJ013179 to KJ013194. To understand the genetic origin of Ca/GX/L1/2013 and Ca/GX/L2/2013, they were initially analyzed by BLASTn (http://www.ncbi.nlm.nih.gov/BLAST). Sequences comparisons revealed that six gene segments of two H3N2 CIVs had highest homology with the corresponding genes of swine H3N2 influenza viruses (SIVs) from the 2000s, including HA, PB2 and PA genes (99.8–99.9%) of swine/Henan/1/2010 (H3N2), PB1 and NP genes (99.8%) of swine/Shandong/3/2005, and M genes (99.8%) of swine/Guangdong/L22/2010 (H3N2), but NA and NS genes were most closely related to the corresponding gene from swine H1N2 influenza virus, swine/Zhejiang/01/2008 (H1N2) (99.7% and 100%, respectively), which is a reassortant of human-like H3N2 viruses, seasonal human H1N1 viruses and classical H1N1 swine viruses (Table 2). These results demonstrated that these two isolates most likely originated from H3N2 swine influenza viruses.

**Table 2:** **Sequence identity of genes of the two H3N2 CIVs isolated in Guangxi to related sequence in GenBank**

**Genes**	**Virus with highest identity**	**Identities** ^**a**^ **(%)**	**GenBank accession No.**	**Influenza virus lineage**	**Common amino acid changes compared to Moscow/10/99 strain** ^**b**^
HA	A/swine/Henan/1/2010(H3N2)	99.9	KF277766	human	T192I, V196A, V226I, **K239N(L2)**
NA	A/swine/Zhejiang/01/2008(H1N2)	99.7	JX138511	human	**S44P**, **V240I**, L370S, **V398I**, I464L
PB1	A/Swine/Shandong/3/2005(H3N2)	99.8	EU116037	human	E75D, **R189K**, **G354R(L2)**, **F512L(L1)**, **M718L(L2)**
PB2	A/swine/Henan/1/2010(H3N2)	99.6	KF541238	human	E241D, F600L, T745A
PA	A/swine/Henan/1/2010(H3N2)	99.8	KF541239	human	Q346K
NP	A/Swine/Shandong/3/2005(H3N2)	99.8	EU116041	human	M105I, **I119T(L1)**, **R195G(L1)**, **251I(L1), A251V(L1)**,D290N, K357R, **A493T**
M	A/swine/Guangdong/L22/2010(H3N2)	99.8	JX494713	human	M1: S275P; M2: **G184R(L1)**, **G199R(L1)**
NS	A/swine/Zhejiang/01/2008(H1N2)	100	JX138513	human	NEP: V82A

^a^The results of identities were dependent on BLASTn analysis.

^b^bold font letter represent the amino acid substitution found in the gene segment of two H3N2 CIVs.

To characterize more precisely the genetic origin of these two H3N2 CIVs, the phylogenetic trees of eight gene segments (HA, NA, NP, PA, PB1, PB2, M, NS) were constructed using MEGA 5.2. Compared with the related reference nucleotide sequences available in GenBank, it was found that canine influenza viruses were mainly divided into human/swine, avian/canine, pdm09-like/canine, and equine/canine lineages based on HA gene (Figure 1a). The two CIVs under study, clustered together with swine H3N2 and human H3N2 (Moscow/10/99) viruses in a human/swine group. Based on whole genome sequence analysis, these two H3N2 CIVs fell into the human/swine lineage, and were clustered within the Moscow/99-like sublineages. Compared with Moscow/10/99, sequence homology reached >99% in each of the eight gene segments. They differed from current circulating human isolates (Maryland/05/2013) or human H3N2 variants (Iowa/07/2011) in the HA gene phylogenetic tree (Figure 1a). In fact, in the late 1990s, the Moscow/99-like H3N2 influenza viruses were introduced into the pig population and maintained transmission in pigs in China [17]. These results indicate that human-like H3N2 swine influenza viruses might cross the species barrier and transmit to dogs.

**Figure 1:**
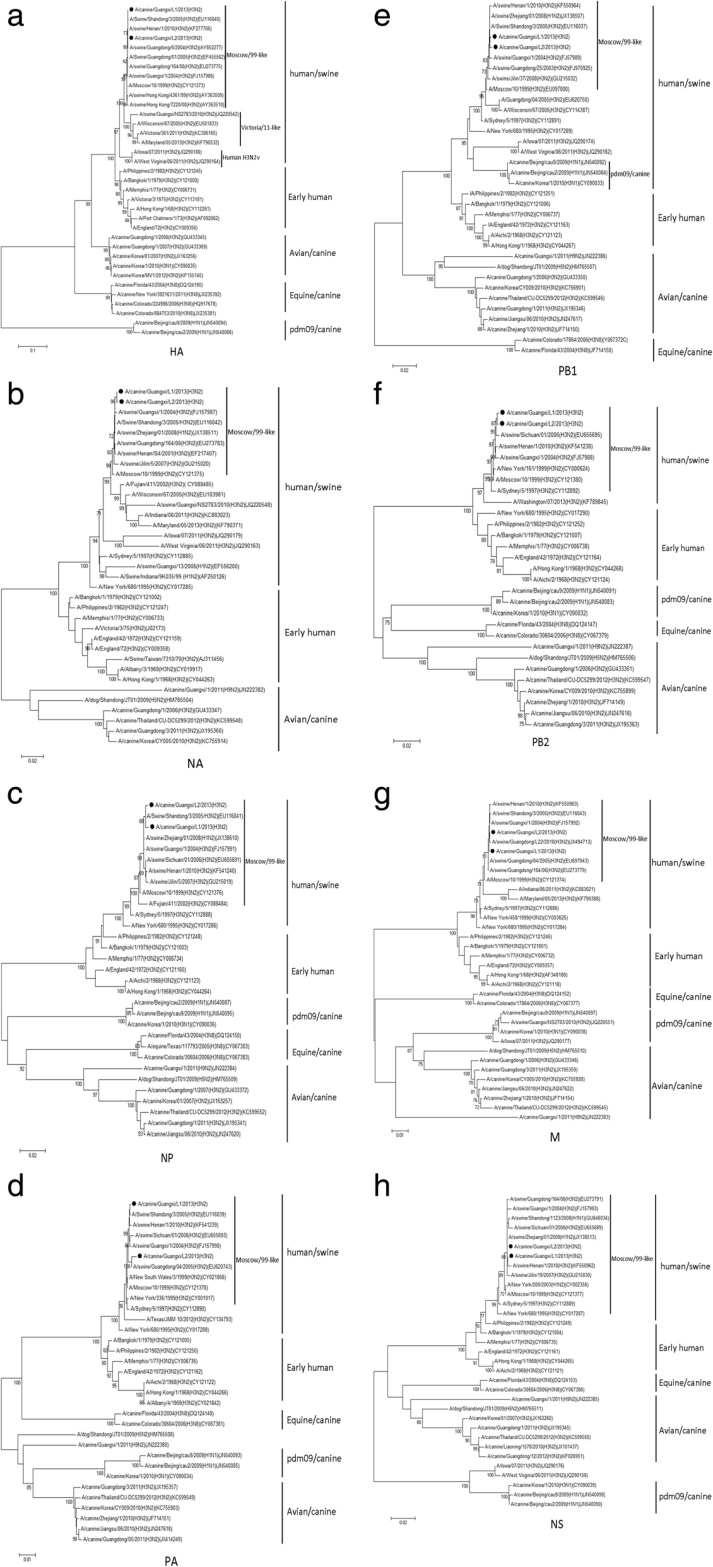
**Phylogenetic trees of eight gene segments of two H3N2 CIVs and reference isolates.** The trees were generated with the MEGA 5.2 program using neighbor-joining analysis. The bootstrap percentages are shown above the nodes that were supported in >70% of 1000 replicates. The viruses isolated in our study are shown in black round circles. The phylogenetic trees are shown in **(a)** HA gene, **(b)** NA gene, **(c)** NP gene, **(d)** PA gene, **(e)** PB1gene, **(f)** PB2 gene, **(g)** M gene, and **(h)** NS gene.

The deduced amino acid sequences of two H3N2 CIVs were compared with those of early human, 1990s human or 2000s swine isolates and contemporary human isolates (Figure 2). Compared with Moscow/10/99, receptor-binding sites of these CIVs were highly conserved at the positions of Y98, W153, H183 and S228 (Figure 2). However, the region of antigenic site B2 was variable, and most swine influenza viruses and our two H3N2 CIVs possessed I192T, V194L and A196V substitutions. These changes could increase the possibility of interspecies transmission between pigs and dogs. Here, our canine isolates encoded isoleucine (I) at position 226 and serine (S) at position 228, which were characteristic for affinity to α2,6-NeuAcGal receptor [18,19]. Besides, canine influenza viruses possessed ten potential N-linked glycosylation sites in HA1, at position 8–10, 22–24, 38–40, 63–65, 122–124, 126–128, 133–135,165-167,246-248 and 285–287.

**Figure 2:**
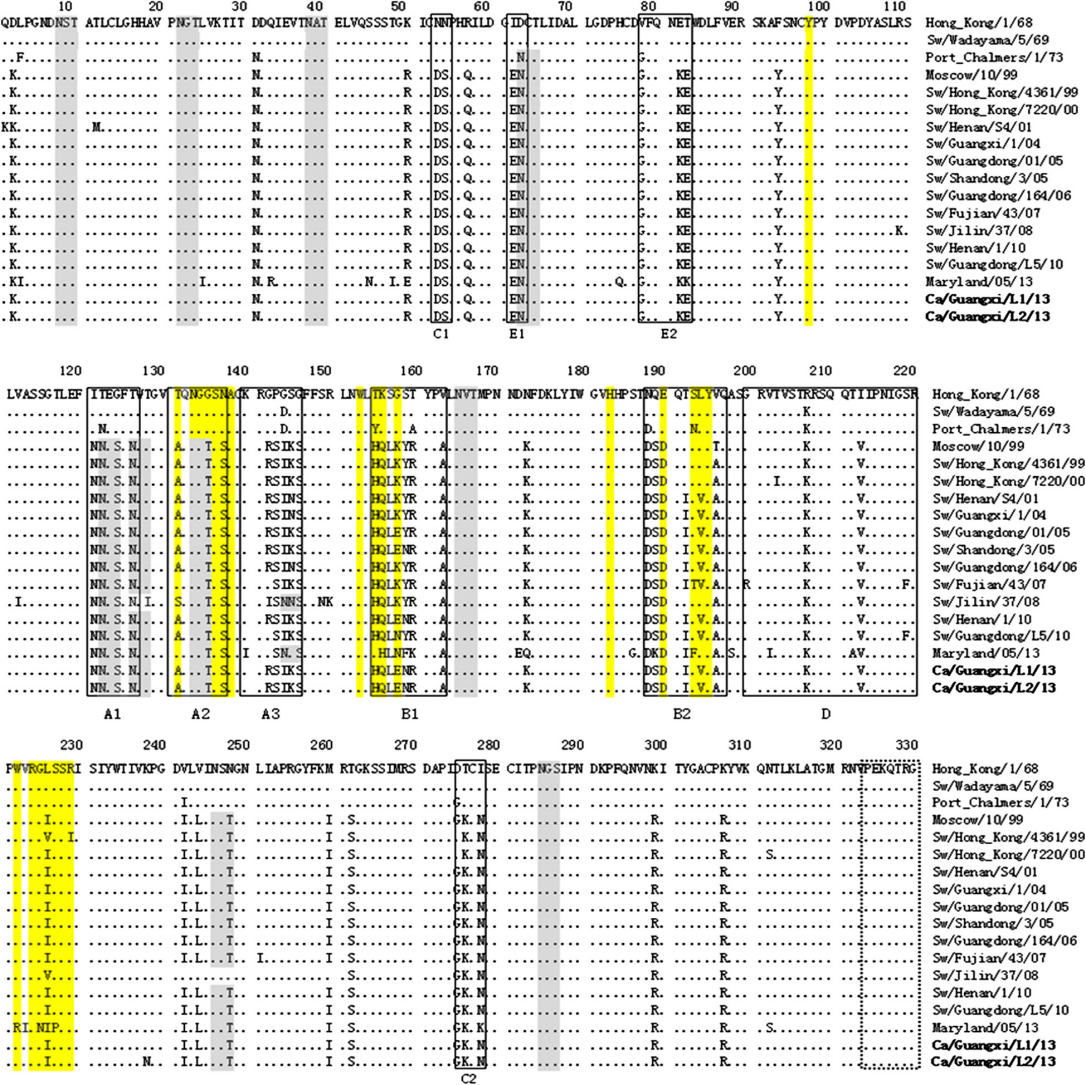
**Alignment of HA1 amino acid sequences of two H3N2 CIVs and representative H3N2 human and swine influenza viruses.** Antigenic sites are indicated by solid boxes (lower case letters indicate discrete antigenic sites), residues in yellow denote the receptor-binding sites, and residues in grey represent the potential glycosylation sites. The cleavage sites are in the dotted box.

There were no deletions in the neuraminidase (NA) stalk region. However, S44P, V240I and V398I substitutions were found in the NA protein of both CIVs. Besides, substitution R189K in PB1 and A493T in NP genes were present in CIVs differed from swine or human influenza viruses (Table 2). Further experiments are required to explore whether these mutations will help human-like H3N2 viruses adapt to dogs.

A total of 315 canine blood samples were separated by centrifugation at 2000 rpm for 10 min and the supernatants were collected into Eppendorf tubes and stored at −20°C, for testing the antibody titer against H3N2 CIVs by hemagglutination inhibition (HI), as previously described [11]. An HI titer ≧40 was considered positive. All tested samples were negative (data not shown). A previous serological survey showed only a 1.2% seropositive rate of human H3N2 influenza viruses in dogs [11]. Ramirez-Martinez, et al. also reported that the seroprevalence of human influenza in dogs only reached 0.9% [10]. All these results suggest that the number of dogs infected with H3N2 human influenza viruses seems to be limited, which means that human influenza viruses have not spread or established themselves in the canine population yet. However, dogs possess specific receptors for human (α2,6-NeuAcGal) and avian (α2,3-NeuAcGal) viruses [20,21], which implies that dogs can be regarded as intermediate hosts that can become co-infected with different subtypes of influenza viruses. Furthermore, pet dogs share the same environment with humans and are in close contact with their owners, therefore increasing the opportunities for pet dogs to be exposed to human influenza viruses.

Emergence of human-like H3N2 influenza viruses in pet dogs, with or without any clinical signs, raises further concerns about whether the viruses have crept into the dog population, and whether novel reassortant influenza viruses will emerge from infection of pet dogs that pose a potential threat to public health. Continued surveillance for influenza viruses in dogs will become essential.
